# Effect of Demecolcine-Assisted Enucleation on the MPF Level and Cyclin B1 Distribution in Porcine Oocytes

**DOI:** 10.1371/journal.pone.0091483

**Published:** 2014-03-13

**Authors:** Suo Li, Jin-Dan Kang, Jun-Xue Jin, Yu Hong, Hai-Ying Zhu, Long Jin, Qing-Shan Gao, Chang-Guo Yan, Cheng-Du Cui, Wen-Xue Li, Xi-Jun Yin

**Affiliations:** 1 Department of Animal Science, College of Agriculture, Yanbian University, Yanji, China; 2 Department of veterinary medicine, College of Agriculture, Yanbian University, Yanji, China; Baylor College of Medicine, United States of America

## Abstract

Demecolcine (DEM) treatment of oocytes induces formation of a membrane protrusion containing a mass of condensed maternal chromosomes, which can be removed with minimal damage prior to somatic cell nuclear transfer (SCNT). However, the effect of this method on the distribution of maturation-promoting factor (MPF) in porcine oocytes has not been reported. Here, the level of MPF and the distribution of cyclin B1 were assessed in porcine oocytes following DEM treatment. In addition, the efficiencies of DEM-assisted and mechanical enucleation were compared, as were the development (*in vitro* and *in vivo*) of these oocytes following SCNT. MPF was uniformly distributed in oocytes that had been treated with 0.4 μg/ml DEM for 1 h. Immunofluorescence microscopy showed that in untreated oocytes, cyclin B1, the regulatory subunit of MPF, accumulated around the spindle, and was lowly detected in the cytoplasm. DEM treatment disrupted spindle microtubules, induced chromosome condensation, and reduced the level of cyclin B1 in the nuclear region. Cyclin B1 was uniformly distributed in DEM-treated oocytes and the level of MPF was increased. The potential of embryos generated from DEM-treated oocytes to develop *in vivo* was significantly greater than that of embryos generated from mechanically enucleated oocytes. This is the first study to report the effects of DEM-assisted enucleation of porcine oocytes on the distribution of cyclin B1. MPF in mature oocytes is important for the development of reconstructed embryos and for efficient SCNT.

## Introduction

Somatic cell nuclear transfer (SCNT) technology has been widely used in animal agriculture and biomedical studies. SCNT has been used to produce transgenic animals that can be used as models in which to study human diseases, as well as knockout pigs that can be used for xenotransplantation studies. Somatic cells were recently reprogrammed to generate induced pluripotent stem (iPS) cells by inducing the expression of four genes. It might be possible to use these iPS cells, in combination with SCNT technology, to treat a range of diseases. During the last decade, several mammalian species have been cloned using SCNT [1]–[4], including pigs [4]. Although the successful production of cloned pigs derived from somatic cell nuclei and the number of cloned pigs increases each year. Despite this, the cloning efficiency of SCNT remains generally low. The first piglets cloned from iPS cells were recently produced in China [5].

The production of cloned pigs by SCNT includes several steps. One important step, namely, enucleation of the recipient oocyte, markedly affects the cloning efficiency. Several methods have been described to enucleate porcine oocytes, including mechanical enucleation, point-press enucleation, pol-scope microscopy enucleation, and induced enucleation. In conventional SCNT protocols, oocytes are mechanically enucleated. However, this method may result in excessive loss of cytoplasm [6]–[7] and molecular factors that are important for correct nuclear reprogramming, mitotic spindle assembly, oocyte activation, and embryonic development [8]–[10]. Demecolcine (DEM)-assisted enucleation induces a membrane protrusion containing a mass of condensed maternal chromosomes that can be removed with minimal damage [11]. This simple method has garnered much attention and has been successfully used to clone pigs by SCNT. Furthermore, rabbit fetuses [12], cloned mice [13], cloned cows [14], cloned transgenic cats [15], second-generation cloned cats [16], and miniature pigs [17]–[18] have all been generated using oocytes that were enucleated by DEM treatment. This indicates that DEM-treated oocytes can be used for SCNT, although the mechanisms by which DEM elicits its effects are unclear.

The cyclin-dependent serine/threonine protein kinase maturation-promoting factor (MPF) is a pivotal regulator of meiosis re-initiation and is important for pre-implantation development of mammalian oocytes. MPF is a heterodimer composed of p34/cdc2 (catalytic subunit) and cyclin B (regulatory subunit). MPF activity is high in metaphase-I and -II (MII) mammalian oocytes. Fertilization [1], [2] and parthenogenetic activation [3] induce inactivation of MPF in mature oocytes. However, specific mechanisms must serve to maintain MPF activity in oocytes. To improve enucleation without reducing MPF activity, attempts have been made to increase MPF activity using drugs. Kubiak *et al.* [19] demonstrated that treatment of mouse MII oocytes with nocodazole increases MPF activity. Wu *et al*. [20] reported that MPF activity in goat oocytes is significantly increased following DEM treatment. In mice, the majority of active MPF is localized at the metaphase plate and is therefore not present in enucleated oocytes, which are used as recipient cytoplasts during nuclear transfer [21]. Most studies in mammals suggest that drug treatments can maintain high MPF activity in mature oocytes. Oocytes enucleated using DEM have developed into live piglets; however, the effect of DEM treatment on MPF activity in enucleated porcine oocytes has not been examined.

The present study investigated the effect of DEM treatment on the level of MPF and the distribution of cyclin B1 in mature porcine oocytes. DEM-assisted enucleation was compared with mechanical enucleation in terms of the ability of embryos to develop normally following SCNT.

## Materials and Methods

### Source of Reagents

All chemicals were purchased from Sigma-Aldrich (St. Louis, MO, USA), unless otherwise stated. The experimental procedures were approved by the Animal Care and Use Committee of Jilin University and were in accordance with the animal welfare guidelines of the US National Institutes of Health. The permit number is SYXK(Ji)2010-0011. Ovaries of pigs were collected from a local slaughterhouse. Laboratory animals (a Wuzhishan miniature pig and Large White/Landrace cross-bred adult estrus sows) were purchased from the Agricultural Science and Technology Institute of Yanbian. Ten multiparous sows on the first day of natural estrus were selected as recipients. All operations were performed in a sterile operating room.

### Oocyte Retrieval and *in vitro* Maturation of Oocytes

Ovaries were collected from a local slaughterhouse, incubated in 0.9% NaCl containing 75 µg/ml penicillin G and 50 µg/ml streptomycin sulfate at 25–37°C, and transported to the laboratory within 2 h of collection. Cumulus-oocyte complexes (COCs) were aspirated from follicles with diameters of 2–5 mm using a 20-gauge needle attached to a 10 ml disposable syringe. COCs of good quality were selected, as determined by their homogeneous granulated cytoplasm and at least three uniform layers of compact cumulus cells. The selected COCs were washed three times in Tyrode’s lactate Hepes-buffered solution containing 0.1% polyvinyl alcohol (PVA). Oocytes were cultured in 4-well plates, with each well containing 500 µl of NCSU-37 medium supplemented with 10% porcine follicular fluid, 0.6 mM cysteine, 1 mM dibutyryl cyclic adenosine monophosphate (dbcAMP), 0.1 IU/ml human chorionic gonadotropin (hCG), and 0.1 IU/ml pregnant mare serum gonadotropin for 20–22 h, followed by culture without dbcAMP and hCG for another 18–24 h at 38.5°C in a humidified atmosphere of 5% CO_2_ in air, as previously reported [4]. Mature eggs that had formed the first polar body were collected.

### Donor Cell Culture

Ear fibroblasts were isolated from the tissue of an adult Wuzhishan miniature pig and cultured in a mixture of Dulbecco’s modified Eagle’s medium and Ham’s F-12 medium (Gibco, BRL, Grand Island, NY, USA) supplemented with 10% (v/v) fetal bovine serum in 5% CO_2_ in air at 38°C. Cells between passages 4 and 8 were used as donors for SCNT. A single cell suspension was prepared by standard trypsinization immediately before SCNT.

### Nuclear Transfer

A single donor cell was injected into the perivitelline space of each egg and electrically fused using two direct current (DC) pulses of 150 V/mm for 50 µsec in 0.28 M mannitol supplemented with 0.1 mM MgSO_4_ and 0.01% PVA. Fused eggs were cultured in NCSU-37 medium for 1 h, and then in medium supplemented with 2 mmol/L 6-dimethylaminopurine for 4 h. The reconstructed eggs were activated by two DC pulses of 100 V/mm for 20 µsec in 0.28 M mannitol supplemented with 0.1 mM MgSO_4_ and 0.05 mM CaCl_2_. Activated eggs were cultured in NCSU-37 medium for 6 days in an atmosphere of 5% CO_2_ and 95% air at 38.5°C. After culturing, blastocysts were placed onto a drop of glycerol containing 10 µg/ml Hoechst 33342 on microscope slides. A coverslip was placed on top of the blastocysts and the edge was sealed with nail polish. The nuclei were counted.

### Embryo Transfer

Ten multiparous sows on the first day of natural estrus were selected as recipients. The animals were anesthetized by intravenously injecting ketamine at the ear edge. A ventral midline incision was made. Cloned embryos were transferred to one or both oviducts in each sow (200–250 embryos implanted per oviduct) within 1 day of the onset of estrus. Pregnancy was diagnosed on Day 23–26 (SCNT was performed on Day 0). Fetus development was checked every 2 weeks by ultrasound examination. Cloned piglets were delivered by eutocia on Day 114–118 of gestation.

### MPF Assay

Oocytes were washed several times in Ca^2+^-free phosphate buffered saline (PBS) containing 0.1% PVA, placed in tubes containing 15 μl of radioimmunoprecipitation assay (RIPA) buffer supplemented with a protease inhibitor cocktail (Roche), and stored at –80°C. Samples were vortexed on ice for 4–5 min, and then centrifuged at 4°C at 12,000 rpm for 15 min. The supernatant was collected and stored at –20°C until use. The level of MPF was determined using the Porcine MPF ELISA Kit (Kexing, China) following the manufacturer’s protocol. In brief, the wells of a microtiter plate were coated with a purified anti-porcine MPF antibody, and 10 µl of the oocyte extract was then added to each well, followed by 40 µl of dilution buffer (i.e., the sample was diluted 5-fold). Plates were incubated for 30 min at 37°C in the dark. The wells were washed thoroughly with buffer six times, and 50 µl of a horseradish peroxidase conjugate reagent was added to each well, except the blank well. Samples were incubated for 30 min at 37°C, and then washed thoroughly. The TMB substrate solution was added to each well, the plate was incubated for 15 min at 37°C in the dark, and the reaction was terminated by adding 50 µl of sulfuric acid. The color change was spectrophotometrically measured at 450 nm within 15 min of the reaction being terminated. The concentration of MPF in the sample was determined using a standard curve.

## Experimental Design

### Experiment 1: Effects of DEM Treatment on the Level of MPF in MII Oocytes

The effects of DEM treatment for various amounts of time on the level of MPF were examined. Oocytes were cultured in NCSU-37 containing 0.6 mM cysteine, 4 mg/ml bovine serum albumin (BSA), and DEM for 0.5, 1, 2, or 3 h. The optimal concentration of DEM to induce ooplasmic protrusions in porcine oocytes is 0.4 µg/ml [11], [22]. Control oocytes were cultured in medium lacking DEM. The level of MPF in oocytes (30 per treatment group) was determined.

### Experiment 2: Effects of DEM Treatment on the Distribution of Cyclin B1 in MII Oocytes

#### Oocyte bisection

Mature oocytes that had formed the first polar body were cultured in medium supplemented with 0.4 μg/ml DEM and 0.05 M sucrose for the optimal amount of time (determined in experiment 1). Sucrose was used to enlarge the perivitelline space. Oocytes were bisected by the extrusion method using compression with a blunt pipette tip. To determine whether chromosomes were aligned at the metaphase plate, oocytes were stained with Hoechst 33342, photographed, and washed three times in PBS. The oocyte halves were collected less than 30 min after bisection. Control oocytes were cultured in medium without supplements and bisected by removing half the volume of cytoplasm. For each treatment, the level of MPF was assayed in 25 whole oocytes or 50 oocytes halves.

#### Immunofluorescence microscopy

Immunofluorescence microscopy was performed on whole mounts. DEM-treated or untreated MII oocytes were fixed with PBS containing 4% paraformaldehyde for at least 30 min at room temperature. Cells were permeabilized with PBS containing 1% Triton X-100 for 10 min at 4°C, blocked in 1% BSA for 1 h, and incubated overnight at 4°C with an anti-cyclin B1 antibody diluted 1∶50 in blocking solution. After three washes in PBS containing 0.1% Tween 20 and 0.01% Triton X-100 for 5 min each, oocytes were labeled with FITC-conjugated goat anti-rabbit IgG diluted 1∶100 in blocking solution for 45 min at 37°C in the dark. Samples were incubated with 10 µg/ml Hoechst 33342 for 10 min to label DNA. Stained oocytes were mounted beneath a coverslip using antifade mounting medium to retard photo-bleaching. Slides were examined using laser-scanning confocal microscopy (Leica TCS SP5) and the appropriate filters to simultaneously excite FITC (cyclin B1 labeling) and Hoechst 33342 (DNA labeling).

### Experiment 3: Efficiencies of DEM-assisted and Mechanical Enucleation of Porcine Oocytes, and the Development of these Enucleated Oocytes Following SCNT

#### DEM-assisted enucleation

**Figure 1:**
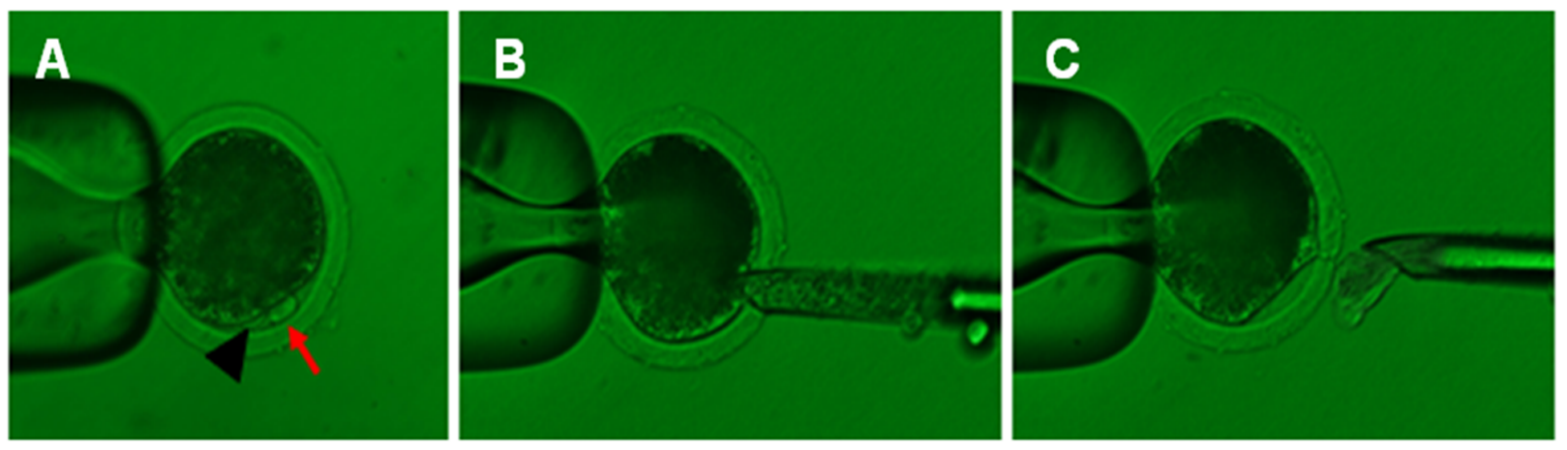
DEM-assisted enucleation method.

DEM-treated oocytes with a protruding membrane were moved to medium supplemented with 5 μg/ml cytochalasin B and 0.4 μg/ml DEM for the optimal amount of time (determined in experiment 1). The protrusion was removed using a beveled pipette, and the first polar body and a portion of the directly underlying cytoplasm were aspirated (Figure 1).

#### Mechanical enucleation

Cumulus cells were removed from newly matured oocytes. Oocytes that had an intact first polar body but that lacked a spontaneous ooplasmic protrusion were micromanipulated in enucleation handling medium. The first polar body and 20–25% of the directly underlying cytoplasm were removed using a glass micropipette (inner diameter of 20 µm).

To determine the efficiency of each enucleation method, the material that was removed during enucleation was incubated with 10 μg/ml Hoechst 33342. Enucleation was confirmed by microscopically examining cytoplasts and karyoplasts. MPF levels were determined before and after enucleation in 50 oocytes per treatment group. To determine the ability of embryos to develop into full-term piglets *in vivo* following DEM-assisted or mechanical enucleation, ten multiparous sows on the first day of natural estrus were used as recipients.

### Statistical Analysis

At least three replicates were performed per experiment. Embryo development data were analyzed using the chi-square test. Data concerning the recipients and the number of cloned piglets that reached adulthood was analyzed using the two-sided Fisher’s Exact test. A *p*-value of less than 0.05 was considered to be significant.

## Results

This study used DEM treatment to induce a membrane protrusion in oocytes and thereby facilitate enucleation (Figure 1).

### Experiment 1: Effects of DEM Treatment on the Level of MPF in MII Oocytes

**Figure 2:**
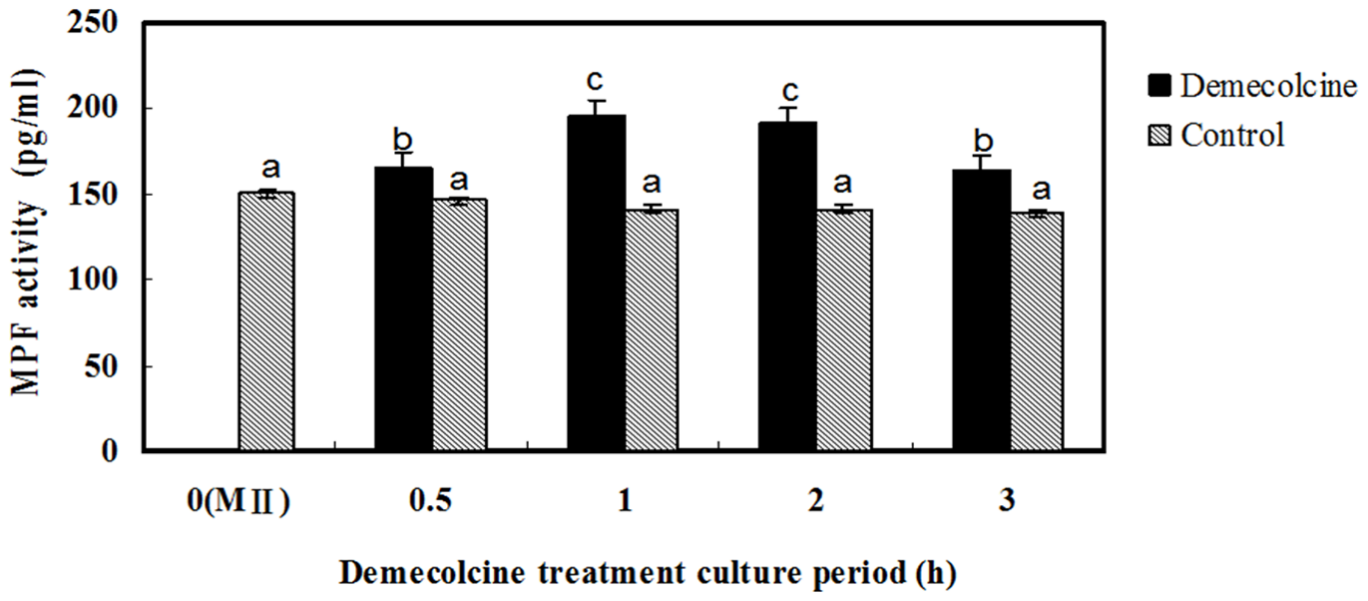
DEM treatment increases the level of MPF in porcine MII oocytes.

Mature denuded MII oocytes were cultured in maturation medium in the presence or absence of 0.4 μg/ml DEM for 0.5, 1, 2, or 3 h, after which the level of MPF was determined. The level of MPF was significantly higher in oocytes treated with DEM for 0.5, 1, 2, or 3 h than in untreated oocytes (Figure 2). The level of MPF was significantly higher in oocytes treated with DEM for 1 or 2 h than in oocytes treated with DEM for 0.5 or 3 h (Figure 2). The level of MPF was highest in oocytes treated with DEM for 1 h and gradually decreased with treatment time thereafter. Therefore, in subsequent experiments, oocytes were treated with 0.4 μg/ml DEM for 1 h.

### Experiment 2: Effect of DEM Treatment on the Distribution of Cyclin B1 in MII Oocytes

**Figure 3:**
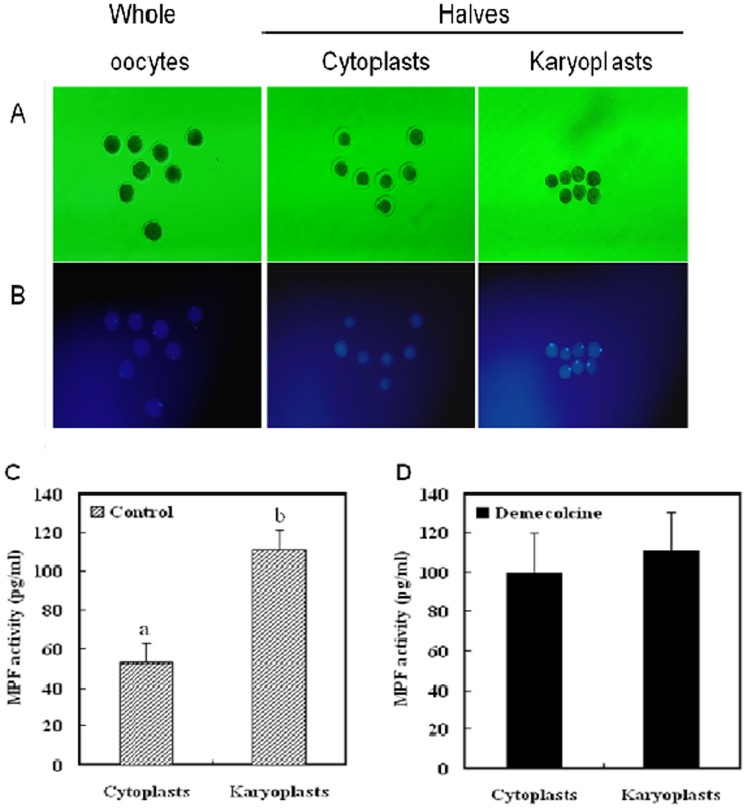
The distribution of MPF markedly differs between porcine oocytes that undergo DEM-assisted enucleation and those that are mechanically enucleated.

To examine the distribution of MPF in oocytes following treatment with 0.4 μg/ml DEM for 1 h, we bisected MII-arrested porcine oocytes and determined the level of MPF in the two halves (karyoplast and cytoplast) (Figure 3). In untreated, mature, denuded porcine oocytes, MPF was unevenly distributed in the cytoplasm; most MPF was localized close to the karyoplast. After oocyte bisection, the MPF level markedly differed between the two halves, with most MPF in the half containing the karyoplast. By contrast, when oocytes were treated with DEM prior to bisection, the MPF level did not differ between the two halves. This result indicates that MPF was uniformly distributed in DEM-treated oocytes, which may owe to depolymerization of the spindle.

**Figure 4:**
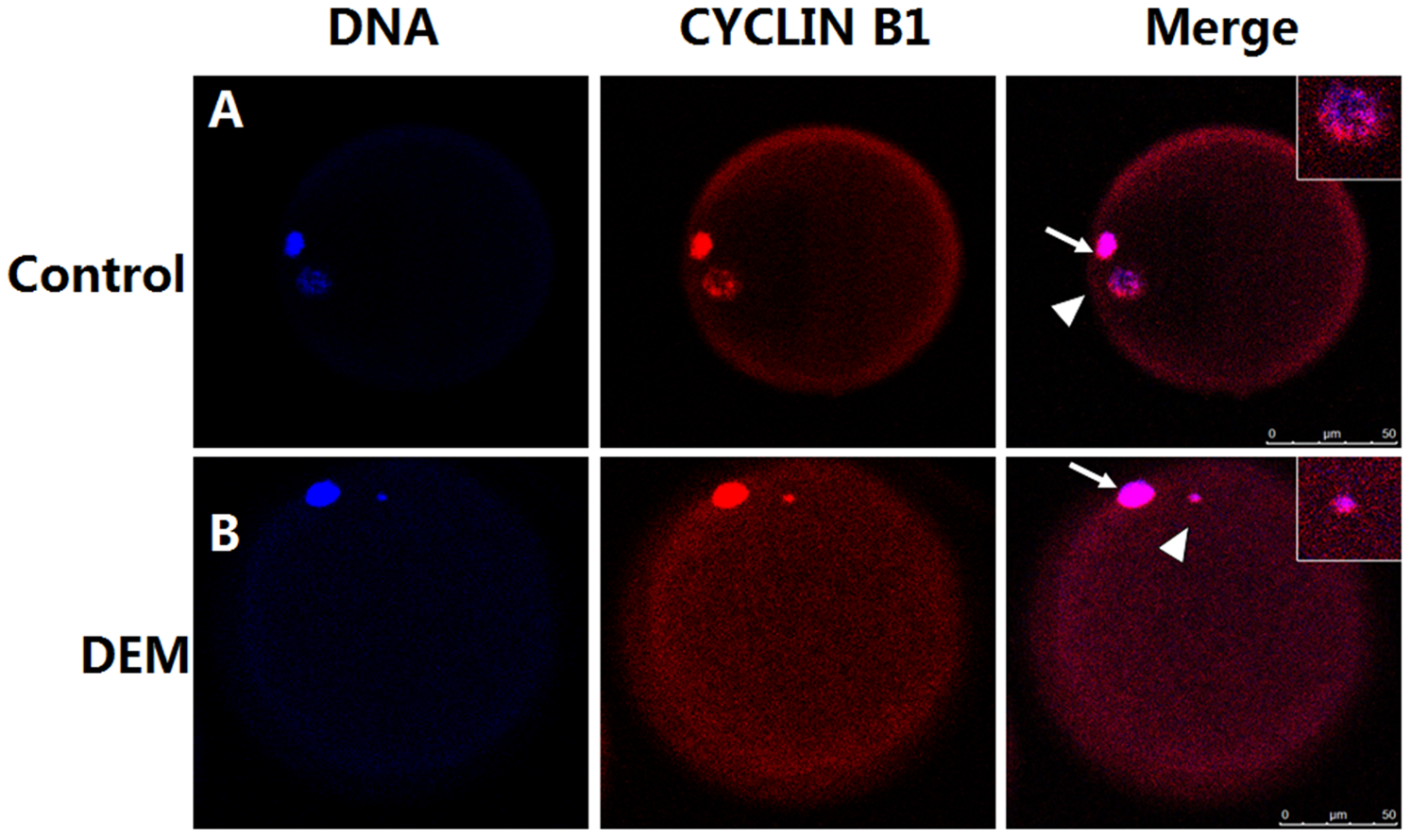
Immunolocalization of cyclin B1 in MII oocytes using confocal microscopy. Bar, 50 µm.

To further observe the distribution of MPF, the localization of cyclin B1, which is the regulatory subunit of MPF, was examined in mature porcine oocytes using immunofluorescence microscopy. In untreated oocytes, cyclin B1 was abundant around the polar body and meiotic spindle, whereas little cyclin B1 was detected in the cytoplast (Figure 4A). After treatment with DEM, the maternal chromosomes were condensed and the level of cyclin B1 was reduced in the nuclear region, but not in the polar body. Overall, the level of cyclin B1 was increased and the protein was uniformly distributed in DEM-treated oocytes (Figure 4B).

### Experiment 3: Efficiencies of DEM-assisted and Mechanical Enucleation, and the Development of these Enucleated Oocytes Following SCNT

Oocytes that had formed the first polar body were cultured in medium supplemented with 0.4 μg/ml DEM and 0.05 M sucrose for 1 h. A membrane protrusion formed that contained a mass of condensed maternal chromosomes, which was easily removed with a small volume of cytoplasm. Immediately after enucleation, the aspirated karyoplast was exposed to ultraviolet (UV) light for 0.1 sec to confirm that it contained the maternal chromosomes. The efficiency of DEM-assisted enucleation of porcine oocytes was significantly higher (P<0.05) than that of enucleation by blind aspiration (Table 1).

**Table 1:** Efficiencies of DEM-assisted and mechanical enucleation of porcine oocytes, and the *in vitro* pre-implantation development of embryos produced from these oocytes.

Enucleationmethod	No. ofoocytes	No. of enucleatedoocytes (%)	No. of SCNTembryos	No. of 2–4-cellembryos (%)	No. ofblastocysts (%)
DEM-assisted	182	179 (98.3)^b^	93	75 (80.6)^a^	15 (16.2)^a^
Mechanical	176	134 (76.1)^a^	85	67 (78.8)^a^	12 (14.1)^a^

a,b Values with different superscripts within the same column are significantly different (P<0.05).

Enucleated porcine oocytes were used as recipient cytoplasts into which pig ear fibroblasts were transferred, and the developmental potential of the resulting embryos was examined. The percentages of embryos that developed to the 2–4-cell and blastocyst stages did not significantly differ between oocytes that underwent DEM-assisted enucleation and those that were mechanically enucleated (Table 1).

**Figure 5:**
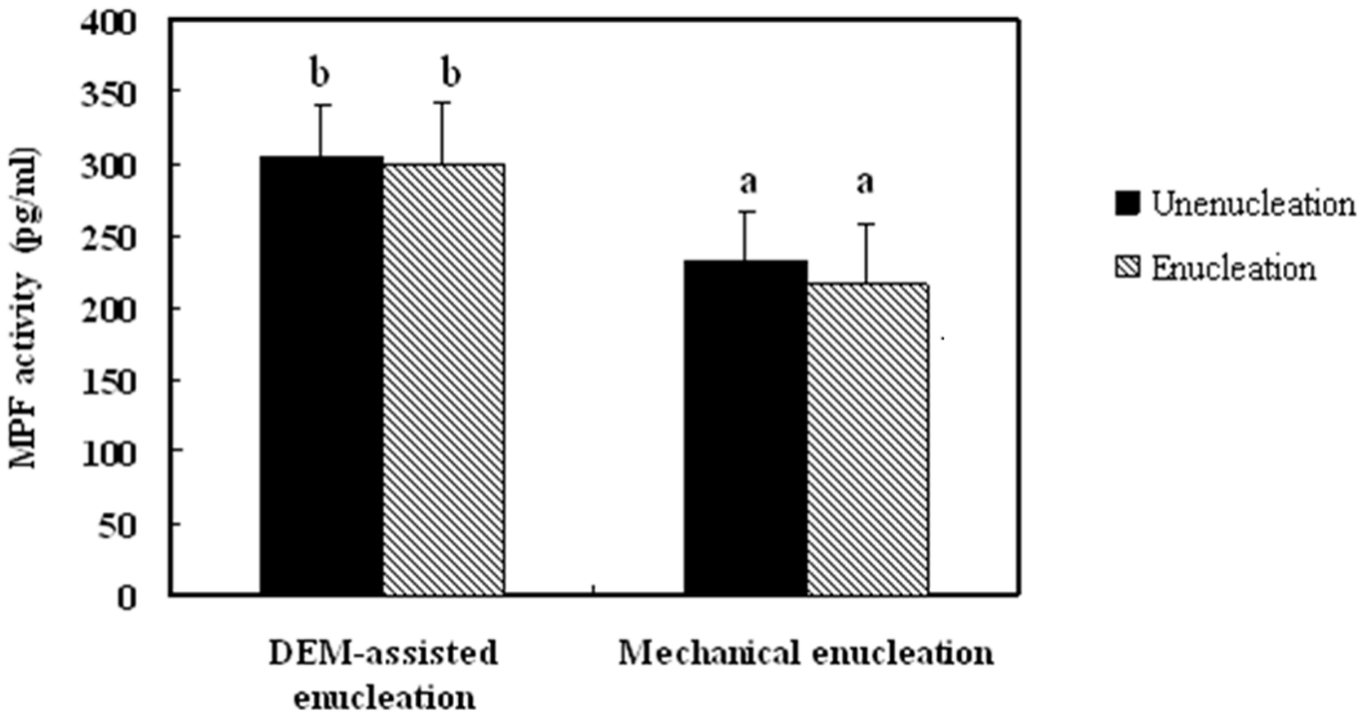
Change in MPF activity in oocytes following DEM-assisted enucleation compared with mechanical enucleation.

We analyze MPF activity in oocytes before and after DEM-assisted or mechanical enucleation. MPF activity in oocytes decreased following mechanical enucleation, although this was not statistically significant, but not following DEM-assisted enucleation (Figure 5). Nevertheless, MPF activity was significantly higher in oocytes that underwent DEM-assisted enucleation than in oocytes that were mechanically enucleated.

**Figure 6:**
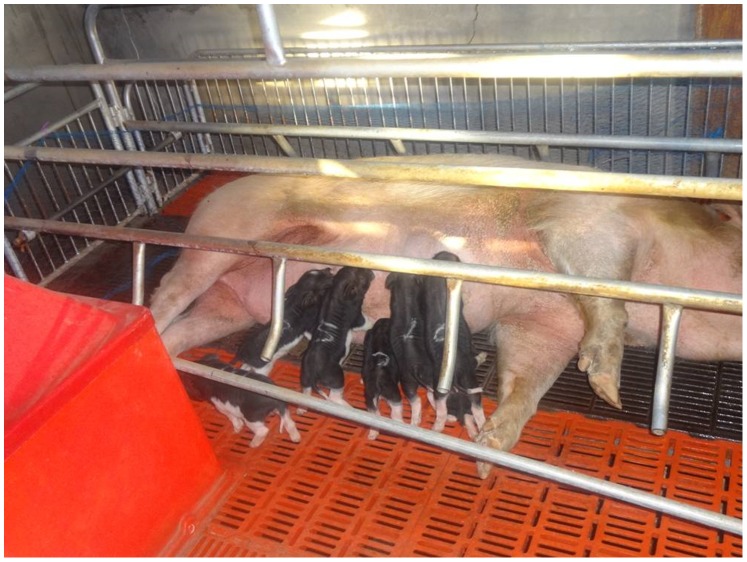
Piglets produced from oocytes that underwent DEM-assisted enucleation.

Embryos generated by SCNT were transferred to surrogates to determine whether DEM-assisted enucleation of oocytes affects the full-term development of embryos. A mean of 217 embryos generated from oocytes that underwent DEM-assisted enucleation were transferred to each of six surrogate sows, of which five (83.3%) became pregnant. After 114–120 days of gestation, four (66.7%) of these sows gave birth to five, seven, five, and eight (one of which died 2 days after birth) cloned piglets, respectively (Figure 6). A mean of 238 embryos generated from oocytes that underwent mechanical enucleation were transferred to each of four surrogate sows, of which three (75.0%) became pregnant. After 114–120 days of gestation, one (25.0%) of these sows gave birth to four cloned piglets, of which two survived to adulthood (Table 2). Thus, the *in vivo* developmental potential of embryos generated from oocytes that underwent DEM-assisted enucleation was markedly greater than that of embryos generated from mechanically enucleated oocytes.

**Table 2:** Full-term development of embryos generated from oocytes that underwent SCNT following DEM-assisted or mechanical enucleation.

Enucleationmethod	No. ofrecipients	No. of recipients thatbecame pregnant (%)	No. of recipients thatunderwent parturition (%)	No. of live clonedpiglets that were born	No. of piglets thatsurvived to adulthood (%)
DEM-assisted	6	5 (83.3)	4 (66.7)^a^	25	24 (96.0)^a^
Mechanical	4	3 (75.0)	1 (25.0)^b^	4	2 (50.0)^b^

a,b Values with different superscripts within the same column are significantly different (P<0.05).

## Discussion

During DEM-assisted enucleation, DEM induces formation of a membrane protrusion that contains a mass of condensed maternal chromosomes, which can be easily removed with minimal damage. This simple method may prove useful for nuclear transfer and has garnered much attention. Here, we assessed changes in the level of MPF and the distribution of cyclin B1 in porcine oocytes following treatment with DEM. We also compared the efficiencies of DEM-assisted and mechanical enucleation, and the development of embryos generated from these enucleated oocytes by SCNT.

Our previous study demonstrated that brief treatment of MII porcine oocytes with DEM produces a membrane protrusion that contains a mass of condensed chromosomes, as seen in DEM-treated bovine oocytes [23] and nocodazole-treated rat oocytes [24]. Although the mechanisms by which DEM elicits its effects are unclear, condensation of maternal chromosomes might cause the protrusion to develop [11]. Yin *et al.* [25] reported that following treatment with 0.4 µg/ml DEM and 0.05 M sucrose for 1, 3, 6, 12, or 24 h, chromosomes were condensed in 100%, 97%, 87%, 96%, and 74% of porcine oocytes, respectively. In this previous study, all oocytes with condensed chromosomes had a protruding membrane, whereas oocytes with dispersed chromosomes did not. Such protrusions are frequently observed in DEM-treated bovine [23] and rabbit [12] oocytes. Sucrose does not affect the formation of a cytoplasmic protrusion containing chromosomes in pig oocytes [26], but does enlarge the perivitelline space. Therefore, the current study investigated the effects of DEM treatment on oocytes. Tetsuya *et al*. [23] demonstrated that DEM treatment of bovine oocytes increases MPF activity, as indicated by an increase in histone H1 kinase activity of up to 30%. Wu *et al.* [20] showed that DEM induces membrane protrusions in goat oocytes by increasing the activities of MPF and mitogen-activated protein kinase via activation of MAD2. Further studies confirmed that MAD2 activates MPF mainly by preventing proteolysis of cyclin B [30]–[32]. Several studies have indicated that treatment of somatic cells with microtubule-interacting agents increases MPF activity [29]–[31], [33]. Although a number of studies have examined somatic cell responses, however, few studies have examined the effects of MPF on oocytes, and it is unknown whether DEM increases MPF activity in pigs. In the current study, a high proportion of mature denuded MII porcine oocytes developed a membrane protrusion following treatment with 0.4 μg/ml DEM for 1 h. The level of MPF was highest in oocytes treated with DEM for 1 h, when it was significantly higher than in untreated oocytes. The level of MPF gradually decreased in oocytes treated with DEM for longer amounts of time. Similar results have been reported in mouse embryos [27], rat oocytes [28], and bovine oocytes [23].

DEM is a microtubule-disrupting agent that depolymerizes microtubules and limits microtubule formation (inactivates spindle fiber formation). The destruction of spindles by DEM inhibits degradation of cyclin B1 [34], which, in turn, increases MPF activity. Changes in the level of cyclin B1 correlate with changes in MPF activity [35]. In the current study, MII porcine oocytes were bisected to examine changes in the distribution of MPF following DEM treatment. MPF was unevenly distributed in the cytoplasm of mature denuded porcine oocytes, and the level of MPF was high in the karyoplast. These results are consistent with a previous study of mouse oocytes [19], and indicate that MPF is predominantly associated with the spindle. By contrast, when oocytes were treated with 0.4 μg/ml DEM for 1 h, MPF activity did not markedly differ between the karyoplast and cytoplast, indicating that MPF was homogenously distributed. To further observe the distribution of MPF, cyclin B1 was examined in mature oocytes using immunofluorescence microscopy. In untreated oocytes, cyclin B1 was accumulated around the meiotic spindle, and was lowly detected in the cytoplasm. In oocytes treated with DEM for 1 h, maternal chromosomes were condensed, the level of cyclin B1 was reduced in the nuclear region, but not in the polar body, and cyclin B1 was homogenously distributed in the cytoplasm. In mouse oocytes, a high level of cyclin B is maintained for several hours following spindle disruption by nocodazole [36], [37].

Activation of MPF depends on the association of p34/cdc2 with cyclin B. Basal levels of p34/cdc2 do not substantially change during *in vitro* maturation of porcine oocytes. However, the level of cyclin B in oocytes tends to increase following *in vitro* maturation [38]. Clute *et al.* [39] reported that the localization of cyclin B1 is extremely dynamic during mitosis. The protein is concentrated at centrosomes and spindle microtubules in organisms ranging from yeast to humans, and is rapidly degraded during late metaphase. Therefore, we examined the localization of cyclin B1 in porcine oocytes by performing immunofluorescence microscopy. DEM treatment disrupted spindle microtubules, induced chromosome condensation, and decreased the level of cyclin B1 in the nuclear region. Overall, in DEM-treated oocytes, the level of cyclin B1 was increased and the protein was uniformly distributed in the cytoplasm. Consequently, MPF activity remained high in oocytes following DEM-assisted enucleation.

The efficiency of DEM-assisted enucleation was significantly higher than that of mechanical enucleation. DEM-assisted enucleation is an attractive method because it induces formation of a membrane protrusion containing a mass of condensed chromosomes. This facilitates removal of maternal chromosomes and produces a competent cytoplast for SCNT. Mechanical enucleation is laborious and technically challenging because it is difficult to locate the meiotic spindle in the oocyte cytoplasm. Therefore, oocytes must often be stained with Hoechst 33342 to label DNA and exposed to harmful UV irradiation [40]–[42]. The pre-implantation development of embryos generated using DEM-assisted enucleation was better than that of embryos generated using mechanical enucleation, and this difference may be owing to the removal of less ooplasm in the former technique. The developmental competence of oocytes following *in vitro* maturation is reportedly related to MPF activity. High MPF activity might promote cytoplasmic maturation and thereby improve the developmental competence of oocytes. In the current study, the level of MPF in oocytes did not decrease following DEM-assisted enucleation, in agreement with a previous report [43], whereas it decreased following mechanical enucleation. The removal of chromosomes from recipient oocytes might specifically remove MPF that is bound to chromosomes or the spindle [25]. Cytoplasts generated via DEM-assisted enucleation and mechanical enucleation markedly differed (data not shown). Whether MPF activity decreases following enucleation may be species-specific. In mice, the majority of active MPF is localized at the metaphase plate and is therefore removed from oocytes by enucleation [44]. This reduction in MPF activity might explain the variable responses of donor nuclei to cytoplasm transfer and differences in the development of the resulting embryos. DEM-assisted enucleation only minimally decreases the cytoplasmic volume of the oocyte and does not reduce the level of MPF, and oocytes enucleated using this method do not need to be stained with Hoechst or exposed to UV radiation. Other chemicals, including nocodazole, etoposide, caffeine, and MG132, have been used to induce or assist oocyte enucleation. Wang *et al.* [45] used caffeine or MG132 to assist enucleation of goat oocytes, and showed that rates of enucleation, cell fusion, and blastula formation are similar among caffeine-, MG132-, and DEM-assisted enucleation, but are significantly lower for mechanical enucleation. Goat embryos produced by caffeine-assisted enucleation have a similar developmental potential to those produced by DEM-assisted enucleation, and similar rates of pregnancy and live births are obtained with each method. However, there are no reports of piglets being generated using caffeine-assisted enucleation.

In the current study, the developmental potential of embryos was examined. The proportions of embryos that developed to the 2–4-cell and blastocyst stages did not significantly differ between oocytes that underwent DEM-assisted enucleation and those that were mechanically enucleated. However, data collected following the transfer of these embryos into surrogates indicated that DEM treatment improves the cloning efficiency. Our laboratory has successfully used DEM-assisted enucleation to produce various cloned animals, including transgenic pigs expressing red fluorescent protein and Wuzhishan minipigs expressing green fluorescent protein. We can conclude that DEM-assisted enucleation is superior to mechanical enucleation for the production of cloned piglets. DEM-assisted and mechanical enucleation of oocytes have been compared in terms of their efficiencies, and the *in vitro* and *in vivo* development of SCNT embryos in several mammalian species other than pigs. Kawakami *et al.* compared embryos generated from oocytes that were enucleated by DEM or nocodazole treatment in terms of their ability to develop into cloned piglets. The first piglets cloned from iPS cells were recently produced in China [5]; however, the cloning efficiency was low. We suggest that DEM-assisted enucleation can improve the cloning efficiency.

In conclusion, this study is the first to demonstrate that DEM-assisted enucleation of oocytes has marked advantages over other enucleation techniques. In DEM-assisted enucleation, MPF is retained in the cytoplast and the amount of cyclin B1 in the nuclear region is decreased. Following DEM-assisted enucleation of oocytes, cyclin B1 is homogenously distributed in the cytoplasm and the level of the protein is increased overall, which corresponds to increased MPF activity. MPF is crucial for the development of reconstructed embryos and for efficient SCNT. Thus, DEM-assisted enucleation appears to be the best method to produce cloned pigs by SCNT.

## Supporting Information

Checklist S1 **The ARRIVE Guidelines Checklist.**
(DOC)

## References

[1] Schnieke AE, Kind AJ, Ritchie WA, Mycock K, Scott AR, *et al.*( (1997) Human factor IX transgenic sheep produced by transfer of nuclei from transfected fetal fibroblasts. Science 278: 2130–3. 9405350 10.1126/science.278.5346.2130

[2] Wakayama T, Perry AC, Zuccotti M, Johnson KR, Yanagimachi R (1998) Full-term development of mice from enucleated oocytes injected with cumulus cell nuclei. Nature 394: 369–74. 9690471 10.1038/28615

[3] Kato Y, Tani T, Tsunoda Y (2000) Cloning of calves from various somatic cell types of male and female adult, newborn and fetal cows. Journal of reproduction and fertility 120: 231–7. 11058438

[4] Baguisi A, Behboodi E, Melican DT, Pollock JS, Destrempes MM, et al (1999) Production of goats by somatic cell nuclear transfer. Nature biotechnology 17: 456–61. 10.1038/8632 10331804

[5] Fan N, Chen J, Shang Z, Dou H, Ji G, Zou Q, et al (2013) Piglets cloned from induced pluripotent stem cells. Cell research 23: 162–6. 23247628 10.1038/cr.2012.176 PMC3541650

[6] Peura TT, Lewis IM, Trounson AO (1998) The effect of recipient oocyte volume on nuclear transfer in cattle. Molecular reproduction and development 50: 185–91. 9590535 10.1002/(SICI)1098-2795(199806)50:2<185::AID-MRD9>3.0.CO;2-G

[7] Wakayama T, Yanagimachi R (1998) Fertilisability and developmental ability of mouse oocytes with reduced amounts of cytoplasm. Zygote 6: 341–6. 9921644 10.1017/s096719949800029x

[8] Simerly C, Dominko T, Navara C, Payne C, Capuano S, et al (2003) Molecular correlates of primate nuclear transfer failures. Science 300: 297. 12690191 10.1126/science.1082091

[9] Miyara F, Han Z, Gao S, Vassena R, Latham KE (2006) Non-equivalence of embryonic and somatic cell nuclei affecting spindle composition in clones. Developmental biology 289: 206–17. 16310175 10.1016/j.ydbio.2005.10.030

[10] Van Thuan N, Wakayama S, Kishigami S, Wakayama T (2006) Donor centrosome regulation of initial spindle formation in mouse somatic cell nuclear transfer: roles of gamma-tubulin and nuclear mitotic apparatus protein 1. Biology of reproduction 74: 777–87. 16407502 10.1095/biolreprod.105.044677

[11] Yin XJ, Tani T, Yonemura I, Kawakami M, Miyamoto K, et al (2002) Production of cloned pigs from adult somatic cells by chemically assisted removal of maternal chromosomes. Biology of reproduction 67: 442–6. 12135879 10.1095/biolreprod67.2.442

[12] Yin XJ, Kato Y, Tsunoda Y (2002) Effect of enucleation procedures and maturation conditions on the development of nuclear-transferred rabbit oocytes receiving male fibroblast cells. Reproduction 124: 41–7. 12090917

[13] Gasparrini B, Gao S, Ainslie A, Fletcher J, McGarry M, et al (2003) Cloned mice derived from embryonic stem cell karyoplasts and activated cytoplasts prepared by induced enucleation. Biology of reproduction 68: 1259–66. 12606420 10.1095/biolreprod.102.008730

[14] Russell DF, Ibanez E, Albertini DF, Overstrom EW (2005) Activated bovine cytoplasts prepared by demecolcine-induced enucleation support development of nuclear transfer embryos in vitro. Molecular reproduction and development 72: 161–70. 16007680 10.1002/mrd.20356

[15] Yin XJ, Lee HS, Yu XF, Choi E, Koo BC, et al (2008) Generation of cloned transgenic cats expressing red fluorescence protein. Biology of reproduction 78: 425–31. 18003942 10.1095/biolreprod.107.065185

[16] Yin XJ, Lee HS, Yu XF, Kim LH, Shin HD, et al (2008) Production of second-generation cloned cats by somatic cell nuclear transfer. Theriogenology 69: 1001–6. 18358524 10.1016/j.theriogenology.2008.01.017 PMC7127140

[17] Miyoshi K, Inoue S, Himaki T, Mikawa S, Yoshida M (2007) Birth of cloned miniature pigs derived from somatic cell nuclear transferred embryos activated by ultrasound treatment. Molecular reproduction and development 74: 1568–74. 17427963 10.1002/mrd.20730

[18] Kang JD, Li S, Lu Y, Wang W, Liang S, et al. (2013) Valproic acid improved in vitro development of pig cloning embryos but did not improve survival of cloned pigs to adulthood. Theriogenology 79: 306–11 e1. 10.1016/j.theriogenology.2012.08.021 23140802

[19] Kubiak JZ, Weber M, de Pennart H, Winston NJ, Maro B (1993) The metaphase II arrest in mouse oocytes is controlled through microtubule-dependent destruction of cyclin B in the presence of CSF. The EMBO journal 12: 3773–8. 8404848 10.1002/j.1460-2075.1993.tb06055.x PMC413659

[20] Wu YG, Zhou P, Lan GC, Gao D, Li Q, et al (2010) MPF governs the assembly and contraction of actomyosin rings by activating RhoA and MAPK during chemical-induced cytokinesis of goat oocytes. PloS one 5: e12706. 20856880 10.1371/journal.pone.0012706 PMC2938347

[21] Fulka J Jr, Ouhibi N, Fulka J, Kanka J, Moor RM (1995) Chromosome condensation activity (CCA) in bisected C57BL/6JxCBA mouse oocytes. Reproduction, fertility, and development 7: 1123–7. 10.1071/rd9951123 8848580

[22] Kawakami M, Tani T, Yabuuchi A, Kobayashi T, Murakami H, et al (2003) Effect of demecolcine and nocodazole on the efficiency of chemically assisted removal of chromosomes and the developmental potential of nuclear transferred porcine oocytes. Cloning and stem cells 5: 379–87. 14733755 10.1089/153623003772032871

[23] Tani T, Shimada H, Kato Y, Tsunoda Y (2006) Demecolcine-assisted enucleation for bovine cloning. Cloning and stem cells 8: 61–6. 16571078 10.1089/clo.2006.8.61

[24] Zernicka-Goetz M, Kubiak JZ, Antony C, Maro B (1993) Cytoskeletal organization of rat oocytes during metaphase II arrest and following abortive activation: a study by confocal laser scanning microscopy. Molecular reproduction and development 35: 165–75. 8100426 10.1002/mrd.1080350210

[25] Yin XJ, Kato Y, Tsunoda Y (2002) Effect of delayed enucleation on the developmental potential of nuclear-transferred oocytes receiving adult and fetal fibroblast cells. Zygote 10: 217–22. 12214802 10.1017/s0967199402002289

[26] Miyoshi K, Mori H, Yamamoto H, Kishimoto M, Yoshida M (2008) Effects of demecolcine and sucrose on the incidence of cytoplasmic protrusions containing chromosomes in pig oocytes matured in vitro. The Journal of reproduction and development 54: 117–21. 18239352 10.1262/jrd.19142

[27] Kato Y, Tsunoda Y (1992) Synchronous division of mouse two-cell embryos with nocodazole in vitro. Journal of reproduction and fertility 95: 39–43. 1625248 10.1530/jrf.0.0950039

[28] Galat V, Zhou Y, Taborn G, Garton R, Iannaccone P (2007) Overcoming MIII arrest from spontaneous activation in cultured rat oocytes. Cloning and stem cells 9: 303–14. 17907941 10.1089/clo.2006.0059

[29] Costa-Borges N, Paramio MT, Santalo J, Ibanez E (2011) Demecolcine- and nocodazole-induced enucleation in mouse and goat oocytes for the preparation of recipient cytoplasts in somatic cell nuclear transfer procedures. Theriogenology 75: 527–41. 21074837 10.1016/j.theriogenology.2010.09.022

[30] Ling YH, Consoli U, Tornos C, Andreeff M, Perez-Soler R (1998) Accumulation of cyclin B1, activation of cyclin B1-dependent kinase and induction of programmed cell death in human epidermoid carcinoma KB cells treated with taxol. International journal of cancer Journal international du cancer 75: 925–32. 9506539 10.1002/(sici)1097-0215(19980316)75:6<925::aid-ijc16>3.0.co;2-1

[31] Huang TS, Shu CH, Chao Y, Chen SN, Chen LL (2000) Activation of MAD 2 checkprotein and persistence of cyclin B1/CDC 2 activity associate with paclitaxel-induced apoptosis in human nasopharyngeal carcinoma cells. Apoptosis : an international journal on programmed cell death 5: 235–41. 11225845 10.1023/a:1009652412399

[32] Homer HA, McDougall A, Levasseur M, Yallop K, Murdoch AP, et al (2005) Mad2 prevents aneuploidy and premature proteolysis of cyclin B and securin during meiosis I in mouse oocytes. Genes & development 19: 202–7. 15655110 10.1101/gad.328105 PMC545877

[33] D’Angiolella V, Mari C, Nocera D, Rametti L, Grieco D (2003) The spindle checkpoint requires cyclin-dependent kinase activity. Genes & development 17: 2520–5. 14561775 10.1101/gad.267603 PMC218146

[34] Nixon VL, Levasseur M, McDougall A, Jones KT (2002) Ca(2+) oscillations promote APC/C-dependent cyclin B1 degradation during metaphase arrest and completion of meiosis in fertilizing mouse eggs. Current biology : CB 12: 746–50. 12007419 10.1016/s0960-9822(02)00811-4

[35] Sakamoto I, Takahara K, Yamashita M, Iwao Y (1998) Changes in cyclin B during oocyte maturation and early embryonic cell cycle in the newt, Cynops pyrrhogaster: requirement of germinal vesicle for MPF activation. Developmental biology 195: 60–9. 9520324 10.1006/dbio.1997.8835

[36] Homer HA, McDougall A, Levasseur M, Murdoch AP, Herbert M (2005) Mad2 is required for inhibiting securin and cyclin B degradation following spindle depolymerisation in meiosis I mouse oocytes. Reproduction 130: 829–43. 16322543 10.1530/rep.1.00856

[37] Lefebvre C, Terret ME, Djiane A, Rassinier P, Maro B, et al (2002) Meiotic spindle stability depends on MAPK-interacting and spindle-stabilizing protein (MISS), a new MAPK substrate. The Journal of cell biology 157: 603–13. 12011110 10.1083/jcb.200202052 PMC2173866

[38] Goudet G, Belin F, Bezard J, Gerard N (1998) Maturation-promoting factor (MPF) and mitogen activated protein kinase (MAPK) expression in relation to oocyte competence for in-vitro maturation in the mare. Molecular human reproduction 4: 563–70. 9665339 10.1093/molehr/4.6.563

[39] Clute P, Pines J (1991) Temporal and spatial control of cyclin B1 destruction in metaphase. Nature cell biology. 1999 1: 82–7. 10.1038/10049 10559878

[40] Wakayama T, Yanagimachi R (1998) Fertilisability and developmental ability of mouse oocytes with reduced amounts of cytoplasm. Zygote 6: 341–6. 9921644 10.1017/s096719949800029x

[41] Simerly C, Dominko T, Navara C, Payne C, Capuano S, et al (2003) Molecular correlates of primate nuclear transfer failures. Science 300: 297. 12690191 10.1126/science.1082091

[42] Miyara F, Han Z, Gao S, Vassena R, Latham KE (2006) Non-equivalence of embryonic and somatic cell nuclei affecting spindle composition in clones. Developmental biology 289: 206–17. 16310175 10.1016/j.ydbio.2005.10.030

[43] Goto S, Naito K, Ohashi S, Sugiura K, Naruoka H, et al (2002) Effects of spindle removal on MPF and MAP kinase activities in porcine matured oocytes. Molecular reproduction and development 63: 388–93. 12237955 10.1002/mrd.90022

[44] Fulka J Jr, Ouhibi N, Fulka J, Kanka J, Moor RM (1995) Chromosome condensation activity (CCA) in bisected C57BL/6JxCBA mouse oocytes. Reproduction, fertility, and development 7: 1123–7. 10.1071/rd9951123 8848580

[45] Wang HL, Chang ZL, Li KL, Lian HY, Han D, et al (2011) Caffeine can be used for oocyte enucleation. Cellular reprogramming 13: 225–32. 21453051 10.1089/cell.2010.0101

